# Dihydromyricetin-Loaded *In Situ* Film-Forming Emulsions: A Eudragit^®^ RS 100-Based Strategy for Controlled Topical Delivery

**DOI:** 10.3390/pharmaceutics18091120

**Published:** 2026-09-06

**Authors:** Chatchaya Ponsuremas, Takron Chantadee, Pratchaya Tipduangta, Siriporn Okonogi, Pimpak Phumat

**Affiliations:** 1 Department of Pharmaceutical Sciences, Faculty of Pharmacy, Chiang Mai University, Chiang Mai 50200, Thailand; chatchaya_p@cmu.ac.th (C.P.); takron.chantadee@cmu.ac.th (T.C.); pratchaya.t@cmu.ac.th (P.T.); okng2000@gmail.com (S.O.); 2 Center of Excellence in Pharmaceutical Nanotechnology, Faculty of Pharmacy, Chiang Mai University, Chiang Mai 50200, Thailand; 3 Innovative Research Group: In Situ Bioactive Delivery System (ISBD), Faculty of Pharmacy, Silpakorn University, Nakhon Pathom 73000, Thailand

**Keywords:** *in situ* film-forming emulsion, *in situ* film-forming system, self-nanoemulsification, Eudragit^®^ RS 100, dihydromyricetin

## Abstract

**Background/Objectives:** Dihydromyricetin (DHM), a poorly water-soluble flavonoid with potent antioxidant and skin-rejuvenating properties, exhibits limited skin permeation. This study aimed to develop and characterize DHM-loaded *in situ* film-forming emulsions (FFE) using Eudragit^®^ RS 100 as the film-forming polymer to enhance skin permeation while minimizing systemic absorption. **Methods:** FFE formulations, comprising a DHM-loaded lipid mixture and a Eudragit^®^ RS 100 polymer solution, were developed using a full-factorial design of experiments varying poloxamer 407 (P407) and octyl cyanoacrylate (O60/20)/N-methyl-2-pyrrolidone (NMP) concentrations to evaluate drying time, elongation at break, water vapor transmission rate (WVTR), and dermal permeation. The optimized formulation was further characterized for physicochemical properties, chemical integrity, drug loading, and release behaviour. **Results:** All formulations were homogeneous and physically stable over 24 h. Drying time was positively influenced by P407 but negatively by O60/20/NMP; P407 reduced dermal permeation, while O60/20/NMP had a biphasic effect without significantly affecting elongation at break and WVTR. The optimized FFE (0.079%*w*/*w* O60/20/NMP, without P407) showed rapid drying (2.68 min), elongation >10%, and self-formed nanoscale emulsion droplets from the lipid mixture. FTIR confirmed component compatibility; XRD revealed DHM conversion from a crystalline to an amorphous state. Compared with a system without lipid, FFE exhibited significantly higher moisture content (*p* < 0.001), a lower swelling index (*p* = 0.021), comparable occlusive factor (*p* = 0.68), and followed Higuchi kinetic release. **Conclusions:** The optimized FFE demonstrated desirable film-forming properties and physicochemical compatibility, offering an innovative formulation strategy with translational potential for topical industries. However, substitution of NMP is recommended for cosmetic formulations.

## 1. Introduction

Dihydromyricetin (DHM) is a potent flavonoid compound reported to possess diverse biological activities, including antioxidant, anti-inflammatory, neuroprotective, and anti-aging properties [1,2]. Interest in incorporating DHM into anti-aging skincare formulations has grown accordingly. This trend is supported by mechanistic evidence demonstrating that DHM exerts its anti-aging effects through the inhibition of DNA methyltransferase 1 (DNMT1), resulting in the hypomethylation of epidermal deoxyribonucleic acid (DNA) in keratinocytes [2]. DHM has been incorporated into conventional cosmetic products, such as serums and creams, with the properties of reduced skin roughness and wrinkle visibility and occupancy, and increased dermal echogenicity [3].

However, DHM presents several challenges for dermal application. Its limited aqueous solubility (0.2–0.32 mg/mL) at 25 °C in cold water) [4] restricts dissolution and bioavailability. Moreover, DHM exhibits pH-dependent instability, undergoing rapid oxidation and degradation under alkaline conditions (pH ≥ 8.0) [5]. These limitations necessitate advanced delivery strategies to enhance dermal penetration and therapeutic efficacy.

A novel dermal delivery system, *in situ* film-forming systems, offers significant potential for enhancing the effectiveness of both dermal and transdermal drug delivery. Several studies have demonstrated that FFS can significantly enhance the dermal and transdermal delivery of active ingredients such as diclofenac when compared to conventional solutions, creams, or gels [6]. This enhancement is attributed to the ability of FFS to form a semi-occlusive barrier that concentrates the drug at the site of application and facilitates permeation. In contrast, conventional topical formulations cannot maintain a sufficient drug concentration gradient across the skin, which is essential for optimal permeation. *In situ* film-forming solution containing propolis has been successfully developed and demonstrates enhanced skin permeation compared to conventional topical propolis solution [7]. Typical components of FFS are active compounds, solvents, film-forming polymers, and skin penetration enhancers. The system contains volatile solvents that facilitate rapid film formation upon application [8], whereas non-volatile solvents prevent drug crystallization and enhance dermal absorption [9,10]. Polymers used in these formulations should possess anticrystallization properties to maintain the drug in an amorphous state following solvent evaporation. Commonly employed polymers include polyvinyl pyrrolidone (PVP), polyethylene glycol (PEG), and hydroxypropyl methylcellulose (HPMC), a copolymer consisting of methacrylic acid and ethyl acrylate. These polymers stabilize the formulation and contribute to film integrity [11].

Acrylate/ammonium methacrylate copolymer, commonly known as Eudragit^®^, is used as a film base for *In situ* film-forming systems. This polymer provides several desirable benefits for topical formulations, including the effective encapsulation of the active compounds and a controlled release property, thereby prolonging the therapeutic effect [12]. Eudragit^®^ RS 100 is a cationic copolymer suitable for FFS on skin. Film formation is performed by solvent evaporation; upon application to the skin, the evaporation of the volatile solvent drives chain entanglement and coalescence of the polymer into a continuous film [8]. Excipients such as octyl cyanoacrylate act as a plasticizing excipient; the monomers in the system polymerize on contact with skin moisture via hydroxyl ions initiating anionic chain polymerization, forming a water-resistant polymeric film that improves adhesive performance on the skin [13]. From a previous study, rapid film formation has been demonstrated by using high ethanol to enhance solvent evaporation, and using dimethyl sulfoxide (DMSO) to improve active compound solubility and film plasticity, ensuring the formation of a smooth and flexible propolis-loaded film [7]. However, a high concentration of ethanol has been reported to alter the lipid structure of the stratum corneum and reduce skin hydration, thereby increasing the risk of skin irritation [14]. Furthermore, DMSO is generally restricted in cosmetic and topical formulations due to concerns regarding cytotoxicity [15]. *In situ* film-forming emulsions (FFE) are another novel system that is an emerging class of emulsion system in which oil droplets are homogeneously dispersed within a polymer matrix and form a coherent film after application. This system is particularly advantageous for formulating lipophilic drugs, as it can improve their physicochemical property and control recrystallization during the solvent evaporation process. Additionally, FFEs can improve drug transport across the skin barrier, leading to enhanced bioavailability and therapeutic performance [16].

According to the advantageous properties of film-forming solutions and film-forming emulsions, this study thus investigates the enhancement of DHM skin permeability using Eudragit^®^ RS 100 as the polymeric base without DMSO for novel topical skin delivery systems. The comparative evaluation of both formulation approaches will elucidate the optimal strategy for maximizing DHM skin bioavailability while minimizing cutaneous irritation potential by reducing ethanol concentration, thereby facilitating the development of next-generation topical dermatological products.

## 2. Materials and Methods

### 2.1. Chemicals

All reagents were of analytical grade. Dihydromyricetin (DHM), Caprylic/capric triglyceride or MCT, propylene glycol, and polyethylene glycol 400 (PEG400) were purchased from My Skin Recipe (Bangkok, Thailand). PEG-40 hydrogenated castor oil, or Eumulgin^®^ CO 40, was obtained from BASF (Ludwigshafen, Germany). Eudragit^®^ RS 100 was kindly donated by Evonik Nutrition & Care GmbH (Essen, Germany). Octyl cyanoacrylate (O60/20) was obtained from Henkel (Düsseldorf, Germany); N-methyl-2-pyrrolidone (NMP) was obtained from LOBA Chemie (Mumbai, India); dimethyl sulfoxide was obtained from Fisher Scientific UK (Loughborough, UK). Poloxamer 407 (P407) was obtained from Sigma-Aldrich, Inc. (St. Louis, MO, USA). Methanol, ethanol, and isopropyl alcohol (IPA) were obtained from QRëC (Auckland, New Zealand). Ethoxydiglycol or Transcutol^®^ CG and Maisine^®^ CC were obtained from Gattefossé (Lyon, France). Polyvinylpyrrolidone K30 (PVP K30) and phosphate-buffered saline were from HiMedia Laboratories LLC (Kennett Square, PA, USA). Phosphoric acid was obtained from Kemaus (New South Wales, Australia). Anhydrous calcium and potassium chloride were purchased from Merck & Co., Inc. (Rahway, NJ, USA).

### 2.2. High Performance Liquid Chromatography (HPLC) Analysis

DHM quantification was performed by an HPLC form Agilent Technologies Inc. (Santa Clara, CA, USA) equipped with a diode array detector (DAD). A chromatographic separation was achieved via a stationary-phase Agilent Eclipse XDB-C18 (5 µm × 4.6 mm × 150 mm) HPLC column. The analysis was done using an isocratic system using a mobile phase containing methanol and 0.05% phosphoric acid in a ratio of 70:30. The mobile phase was filtered using 0.45 µm filter paper and degassed for about 15 min by sonication. The flow rate was set at 0.5 mL/min, and DHM was detected at 292 nm. A sample volume of 10 µL was injected for each analysis. The intact DHM at a concentration range of 3.125–800 µg/mL was subjected to HPLC to construct a linear standard curve.

### 2.3. Development and Optimization of DHM Loaded in In Situ System

The formulation was developed and modified from our previous study [7] using a statistical approach based on mathematical models or equations, specifically employing a full factorial design under the design of experiment (DoE) methodology via Design Expert version 10. A full factorial design was employed to investigate the effects of multiple independent variables, which were the concentrations of P407 and O60/20/NMP, on key response parameters, including drying time, water vapor transmission rate (WVTR), film mechanical strength (elongation at break), and drug permeation, as shown in Table 1. P407 was selected as an independent variable owing to its well-documented thermogelling and viscosity-modifying properties [17], hypothesized to influence film-forming kinetics, mechanical strength, and drug release behavior. O60/20/NMP was likewise selected based on the known plasticizing and adhesive film-forming role of octyl cyanoacrylate, which polymerizes on contact with skin moisture to form a water-resistant polymeric film [13], and was hypothesized to affect drying time and mechanical flexibility.

### 2.4. Preparation of DHM Loaded in In Situ Systems

The *In situ* system with an oil phase, which was called FFE, was prepared by mixing DHM in a lipid mixture with a polymer solution. Firstly, the lipid mixtures modified from Okonogi et al. [20], consisting of 20%*w*/*w* MCT, 30%*w*/*w* Maisine^®^ CC, 40%*w*/*w* Eumulgin^®^ CO 40, and 10%*w*/*w* ethanol, were prepared with some modification adjustments following the previous study [20]; 2.5%*w*/*w* of DHM was then added to the lipid mixture. The polymer part provided from DOE was prepared. Eudragit^®^ RS 100, used as a film-based polymer, was first dissolved in ethanol under continuous stirring until complete swelling and dissolution were achieved. Subsequently, 5%*w*/*w* PG, 1%*w*/*w* Transcutol^®^ CG, 7%*w*/*w* IPA, 1.9%*w*/*w* PEG 400, 3%*w*/*w* PVP K30, and various concentrations of P407 (0–4%*w*/*w*) were mixed. A mixed-solvent system, termed O60/20/NMP, was prepared by combining O60/20 and NMP at a fixed weight ratio of 5:95, as established in our preliminary study, since O60/20 requires NMP as a co-solvent to achieve complete dissolution within the polymer solution. This combined solvent system was subsequently added to the polymer solution at 0–0.5%*w*/*w* and stirred until a uniform dispersion was obtained. All components were mixed in accordance with standard pharmaceutical compounding procedures. The amount of each excipient followed the DoE result. Outer appearance and dependent variables in Table 1 were characterized in all formulations. The optimized FFE formulation that provided the desirable film properties was selected to investigate in comparison with a polymer solution without lipid mixture, which will be called FFS.

### 2.5. Characterization of the FFE Formulations

#### 2.5.1. Visual Analysis

All formulations were visually inspected for outer appearance, clarity, color, and signs of instability after preparation, which indicate preliminary physical compatibility.

#### 2.5.2. Drying Time Evaluation

The drying behavior of the film was measured by applying the FFE onto a Strat-M^®^ membrane (Merck, Rahway, NJ, USA) covering an area of 28.26 mm^2^. The time required for a completely dry film was recorded using a stopwatch. Complete dryness was confirmed by gently touching the film surface to ensure no residue or tackiness remains. Drying duration can be categorized into three levels: short (≤5 min), moderate (5–7 min), and long (>7 min) [21,22]. The target value for film drying time was set at ≤5 min, with formulations meeting this criterion considered acceptable, which is considered indicative of rapid film formation. The experiment was done in triplicate.

#### 2.5.3. Mechanical Properties

The mechanical strength of the DHM-loaded FFE was evaluated by measuring the elongation at break (%) of the dried film using a texture analyzer (TA-XT plus, Stable Micro Systems, Godalmin, UK). All measurements were performed in triplicate.

The percentage of elongation at break was calculated using Equation (1).

(1) $$\mathrm{Elongation}\;\mathrm{at}\;\mathrm{break}\;(\%)=\frac{Increase\;in\;length}{Original\;length}\times 100$$

#### 2.5.4. Water Vapor Transmission Rate (WVTR)

The WVTR properties of the film, which reflect its breathability, were determined using a desiccant method. Briefly, the film sample was securely mounted over the opening of a glass cup filled with 5 g of silica gel. The sealed cup was then placed in a controlled environment chamber set at 32 °C and 70% relative humidity (RH). The cup’s weight was measured every hour for 8 h to monitor water vapor permeation through the film. All measurements were performed in triplicate.

The WVTR rate was calculated using Equation (2) based on weight changes over time [23,24]:

(2) $$\mathrm{WVTR}\;=\;\frac{W_{f}-W_{i}}{T\times A}\;$$

where W_f_ is the final weight, W_i_ is the initial weight, T is the time during which a weight change occurred, and A is the test area (cup mouth area).

### 2.6. In Vitro Drug Permeation Study

The in vitro drug permeation of DHM was assessed using the ReloGO™ automated diffusion system (REVIVO BioSystems, Singapore), with Strat-M^®^ membrane serving as the skin model with a diffusion area of 50.27 mm^2^. The system was maintained at 32 ± 2 °C with a receptor flow rate of 250 µL/min, which drives medium vertically through the membrane to simulate systemic (blood) circulation [25]. A total of 50 µL of the test sample, containing DHM at a concentration of 0.25 mg/50 µL, was applied onto the Strat-M^®^ membrane, and PBS (pH 7.4) was used as the medium. The fractionated medium was collected at 15, 30, 45, 60, 120, 180, 240, 300, 360, 420, and 480 min. At the end of the experiment, the Strat-M^®^ membrane and the residual film were separately sonicated in 1 mL methanol for 10 min to extract any retained drug. All the collected samples were analyzed by HPLC to quantify DHM content. Cumulative DHM permeated into the receiving chambers over time represented drug reaching the systemic compartment, whereas DHM retained within the Strat-M^®^ membrane represented drug that had penetrated the dermal layer without reaching systemic circulation. It should be noted that, in this study, the ‘permeation’ of DHM refers to its ability to penetrate into the dermal skin layer, whereas ‘membrane retention’ refers to the amount of DHM retained within the skin layer.

### 2.7. Chemical Integrity, Compatibility, and Interactions

To analyze potential interactions between dihydromyricetin and excipients in the formulation, spectral analysis was conducted using a Fourier transform infrared spectrometer (FTIR) (470FT-IR, Nicolet Nexus, Waltham, MA, USA) with an attenuated total reflectance (ATR) accessory.

### 2.8. X-Ray Diffraction (XRD) Analysis

The polymorphism of DHM was investigated via an X-ray diffractometer (MiniFlex, Tokyo, Japan). The study was performed in comparison of intact DHM, DHM in oil mixture, DHM in optimized FFE and FFS formulations. Diffractograms were recorded over a 2θ scanning range of 10–60° at a scanning rate of 10°/min with a step size of 0.01°.

### 2.9. Particle Size Analysis

The droplet size and size distribution, expressed as the polydispersity index (PdI), of the emulsion droplets obtained from the FFEs were determined by dynamic light scattering (DLS) technique using a Zetasizer (Malvern Instruments, Worcestershire, UK). Emulsion droplets embedded within the films were recovered by dissolving the films in deionized water before analysis. The DHM-free oil phase dispersed in DI water, the DHM-containing oil phase in DI water, and the DHM-containing oil phase dispersed in 37% ethanol (corresponding to the FFE system) were evaluated for comparison. All measurements were performed in triplicate.

### 2.10. Characterization and Comparison of the Optimized FFE and FFS Film Properties

#### 2.10.1. Occlusive Factor

The occlusive factor, also known as the F-factor, was determined by comparing the reduced water weight in a glass cup covered with filter paper with and without the film after a storage period for 24 h at 32 C° and 50% RH. The difference in water loss was used to calculate the F-factor using Equation (3) [26,27]. All measurements were performed in triplicate.

(3) $$F\text{-}\mathrm{factor}=\frac{\left(Q-B\right)}{Q}\times 100$$

Q is the reduced water weight in the glass beaker without the film, and B is the reduced water weight in the glass beaker with the film.

#### 2.10.2. Moisture Content

The moisture content uptake behavior of the film is useful information to indicate film storage conditions and predict product stability. Moisture content was assessed by measuring the change in weight of the film according to Equation (4) [28,29]. All measurements were performed in triplicate.

(4) $$\mathrm{Moisture}\;\mathrm{content}\;(\%)=\frac{W_{1}-W_{2}}{W_{1}}\times 100$$

W_1_ is the initial weight of the film, and W_2_ is the constant weight of the film after being placed in a desiccator containing anhydrous calcium chloride (0% RH) at room temperature for 24 h.

#### 2.10.3. Moisture Uptake

To obtain the moisture uptake value, a sample of the dry film was placed in a desiccator for 24 h and then exposed to a saturated solution of potassium chloride at 85% RH in another desiccator. The moisture uptake was calculated using Equation (5) [30,31]. All measurements were performed in triplicate.

(5) $$\mathrm{Moisture}\;\mathrm{uptake}\;\%=\frac{W_{2}-W_{1}}{W_{1}}\times 100$$

W_1_ is the initial weight of the film, and W_2_ is the constant weight of the film after exposure to a different relative humidity.

#### 2.10.4. Swelling Index

To evaluate the swelling index, the dry film sample was weighed before and after being immersed in 35 g of distilled water. The sample was typically agitated at around 50 rpm at room temperature for 1 h using a shaking incubator (N-BIOTEK, Bucheon, Republic of Korea). The degree of swelling was then calculated using Equation (6). All measurements were performed in triplicate.

(6) $$\mathrm{Swelling}\;\mathrm{index}\;(\%)=\frac{W_{1}-W_{0}}{W_{0}}\times 100$$

W_0_ is the initial weight of the dry film, and W_1_ is the constant weight of the swollen film after removal of excess distilled water.

#### 2.10.5. Drug Loading and Drug Content

This assessment was done by dissolving the film in a solvent, methanol, and stirring for 10 min at room temperature. The samples were then filtered and analyzed by HPLC. All measurements and sample preparation were performed in triplicate.

The loading capacity and the drug content were calculated using Equations (7) and (8) [32,33,34].

(7) $$\mathrm{Drug}\;\mathrm{loading}\;\mathrm{capacity}\;(\%)=\frac{Weight\;of\;drug\;in\;the\;FFE}{Weight\;of\;the\;FFE\;with\;the\;drug}\times 100$$

(8) $$\mathrm{Drug}\;\mathrm{content}\;(\%)=\frac{Weight\;of\;drug\;in\;the\;FFE}{Weight\;of\;drug\;added\;in\;the\;FFE}\times 100$$

#### 2.10.6. Viscosity Assessment

The viscosity and rheological behavior of the optimized FFE and FFS formulations were evaluated using a rheometer (Brookfield, MA, USA) with a cup-and-bob geometry at a controlled temperature of 25 °C. Measurements were performed under a constant shear rate of 100 s^−1^ for 60 s, collecting 60 data points at 1 s intervals. Results were reported as viscosity (η, Pa·s) and shear stress (τ, Pa). All measurements were performed in triplicate.

### 2.11. Surface Morphology Evaluation of the Film

The surface morphology of the film, including microscopic structure, surface roughness, and homogeneity, was examined by scanning electron microscopy (SEM) (JSM-IT300, JEOL, Tokyo, Japan) [9].

### 2.12. Drug Kinetic Release Study

The drug release kinetic study was conducted using ReloGO^TM^ (REVIVO BioSystems, Singapore), following the same apparatus and method as the drug permeation study, with PBS buffer (pH 7.4) used as the receptor medium. A cellulose membrane with MWCO of 12,000, covering a diffusion area of 50.27 mm^2^ was placed instead of the Strat-M^®^ membrane. A fixed dose of 50 µL of FFE or FFS film (containing 0.25 mg DHM), consistent with the drug permeation study, was applied to the membrane surface. Sample collection was divided into two phases, automatically operated by the ReloGO^TM^ system: phase I, in which samples were collected every 15 min during the first hour, and phase II, in which samples were collected every 60 min for the subsequent 7 h. The medium was maintained at a flow rate of 20 µL/min. Because the ReloGO™ system operates as an automated continuous-flow perfusion apparatus, fresh receptor medium was continuously supplied at 20 µL/min throughout the study, while the effluent, containing released DHM, was continuously withdrawn for sampling. This continuous replenishment inherently maintained sink conditions throughout the experiment.

### 2.13. Statistical Analysis

Data are presented as mean ± standard deviation (S.D.) and standard error (S.E.). Statistical analysis was performed using DOE software version 2, SPSS software version 17.0, and Excel software for Windows. Analysis of variance (ANOVA) was used to assess the main effects and interaction effects of formulation variables on each response. A *p*-value of less than 0.05 was considered statistically significant.

## 3. Results and Discussion

### 3.1. HPLC Analysis

**Figure 1:**
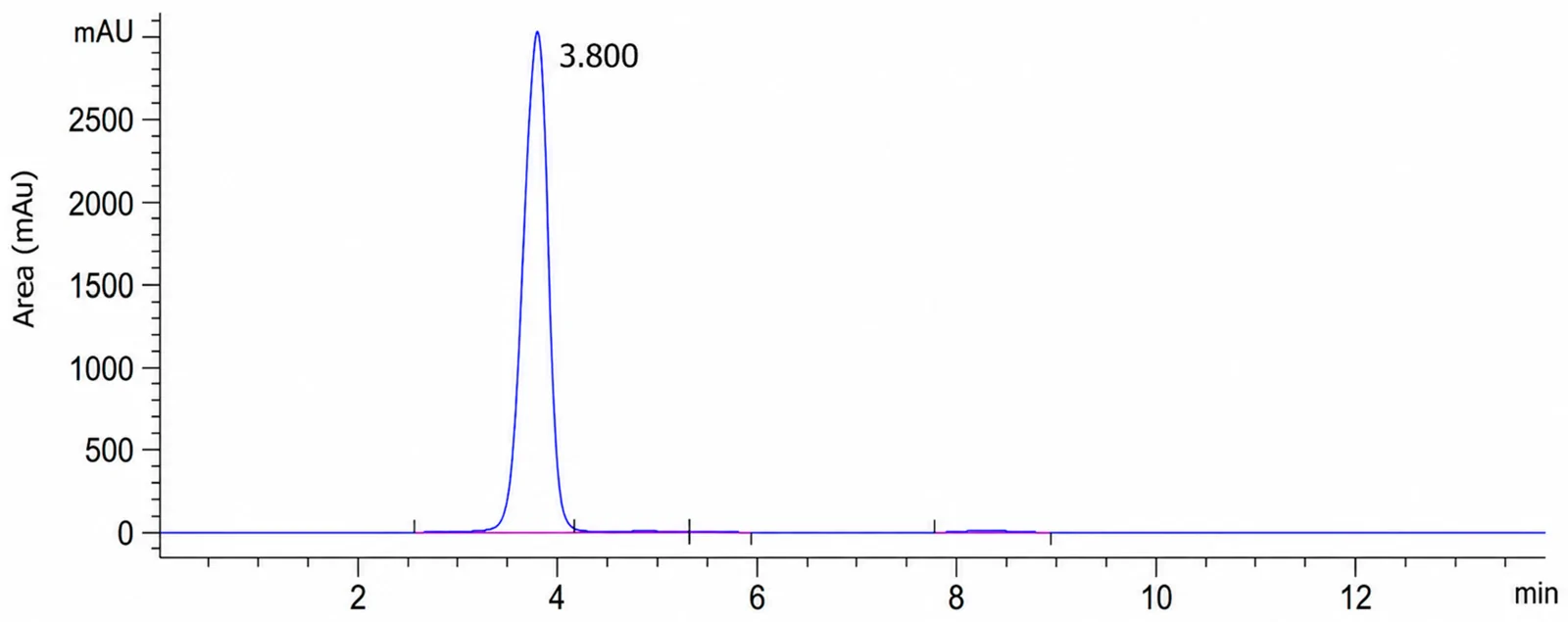
HPLC chromatogram of DHM, analysis at 292 nm.

The HPLC chromatogram shows a well-resolved peak corresponding to DHM at a retention time of 3.91 min (Figure 1). The quantification of DHM was performed using a linear regression equation derived from the standard calibration curve, which was y = 45612x − 513 (R^2^ = 0.993), where y represents the peak area and x represents the DHM concentration.

### 3.2. Developed and Optimized In Situ System

#### 3.2.1. Visual Analysis of FFE Formulations

A DHM-loaded FFE was developed using a full factorial design of experiments approach to identify an optimized formulation for topical delivery. Nine formulations were generated with varying concentrations of P407 and O60/20/NMP, as shown in Table 2. Among these, all nine formulations (F1–F9) exhibited complete dissolution, yellowish homogeneous solutions, and showed no sign of physical incompatibility over 24 h.

#### 3.2.2. Characterization of FFE Formulations for DoE

All the results from this demonstration are demonstrated in Table 2. The drying time of formulations was found to be in the range from 2.84 ± 0.53 to 5.21 ± 0.84 min. As the concentration of P407 increased, the drying time also increased. The same effect was shown in the study of Fakhari [35], since the drying of films formed by P407 is governed by solvent evaporation from the polymer solution or dispersion. Parameters such as polymer concentration can significantly affect the drying process; as the solvent is removed, the structure of the polymer changes and further retards evaporation [36]. Most of the FFE formulations (F1–7, 9) show the desired drying time (less than 5 min), which revealed good *in situ* film-forming properties for topical application [16]. All films were yellowish, clear, homogeneous, smooth, flexible, and conformed to the surface.

WVTR values of all formulations were comparable, with no statistically significant differences observed (*p* = 0.906). This suggests that the modification of formulation variables did not influence the barrier properties of the films, indicating consistent permeability characteristics across all formulations.

Elongation at break was determined to characterize polymeric films for their abrasion resistance and flexibility [37]. It was calculated for each film by using Equation (1). The value was found to be in the range from 111.63 ± 5.94% to 122.02 ± 3.28%. The equation obtained from the DoE analysis showed no statistically significant differences among formulations (*p* = 0.1729), indicating that this model does not adequately predict elongation at break within the tested range and should not be used for predictive purposes. An elongation at break exceeding 10% is generally indicative of adequate film flexibility and conformability to skin movement [18]. This finding is consistent with the study by Wiwat P. et al., in which an *In situ* film showed an elongation at break of 105.6%, indicating good elasticity of the facial mask film [38].

For the permeation study, the cumulative DHM permeation of DHM in all FFE formulations (F1–F9) in the Strat-M^®^ membrane ranged from 362.99 to 592.41 µg/mL, as shown in Table 2. No DHM peak was detected in the receptor medium at any time point, indicating that the values reported in Table 2 reflect DHM retained within the Strat-M^®^ membrane, consistent with dermal permeation rather than transdermal permeation into the receptor phase. Increasing P407 from 0 to 4%*w*/*w* consistently reduced permeation. This reduction is consistent with the gelling behavior of P407, which raises matrix viscosity and diffusional tortuosity, thereby retarding drug release and subsequent membrane permeation [39]. The effect of O60/20/NMP was concentration-dependent and biphasic. In the formulations that contained free P407, a low level (0.10%) of O60/20/NMP enhanced the permeation of DHM, whereas a higher level (0.50%) of O60/20/NMP reduced DHM permeation. This might be attributed to NMP acting as a permeation enhancer [40] at low concentrations of O60/20/NMP. Accordingly, the highest permeation was obtained from F1 (0% P407, 0.10% O60/20/NMP) and the lowest from F6 (4% P407, 0.10% O60/20/NMP).

The relationships between the independent variables and each response were described by the following equations derived from the full-factorial DoE analysis (Equations (9)–(12)):

Drying time (min) = 2.76 + 0.6938A − 0.4583B − 0.0062AB + 0.2217A^2^ + 1.01B^2^
 (Adjusted R^2^ = 0.9910, *p*-value = 0.0007) (9)

WVTR (g/m^2^/8 h) = 7.387 × 10^−6^ − 1.050×10^−6^A − 1.786 × 10^−7^B  (Adjusted R^2^ = 0.7410, *p*-value = 0.0073) (10)

Elongation at break (%) = 116.44 − 3.52A + 1.58B − 1.18AB  (Adjusted R^2^ = 0.3617, *p*-value = 0.1729) (11)

Permeation (µg/mL) = 492.23 − 52.69A − 14.34B + 44.63AB  (Adjusted R^2^ = 0.6521, *p*-value = 0.0413) (12)

where A represents the concentration of P407 (%*w*/*w*), and B represents the concentration of octyl cyanoacrylate/NMP (%*w*/*w*).

**Figure 2:**
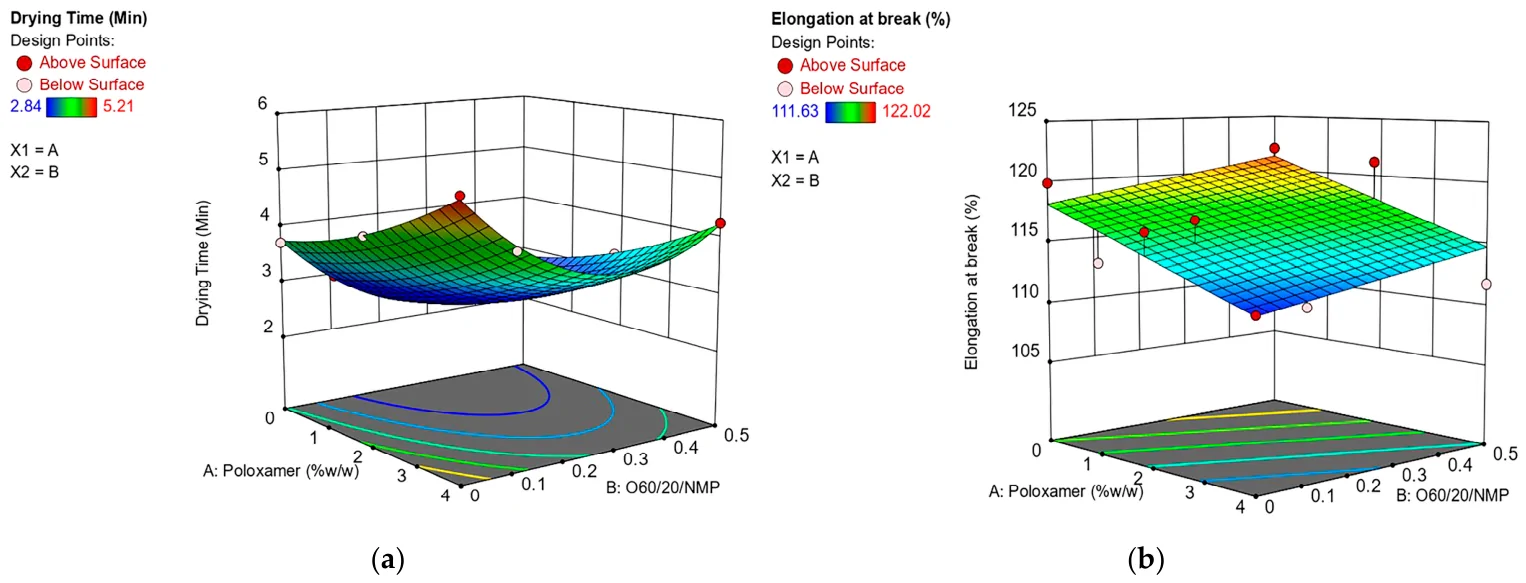

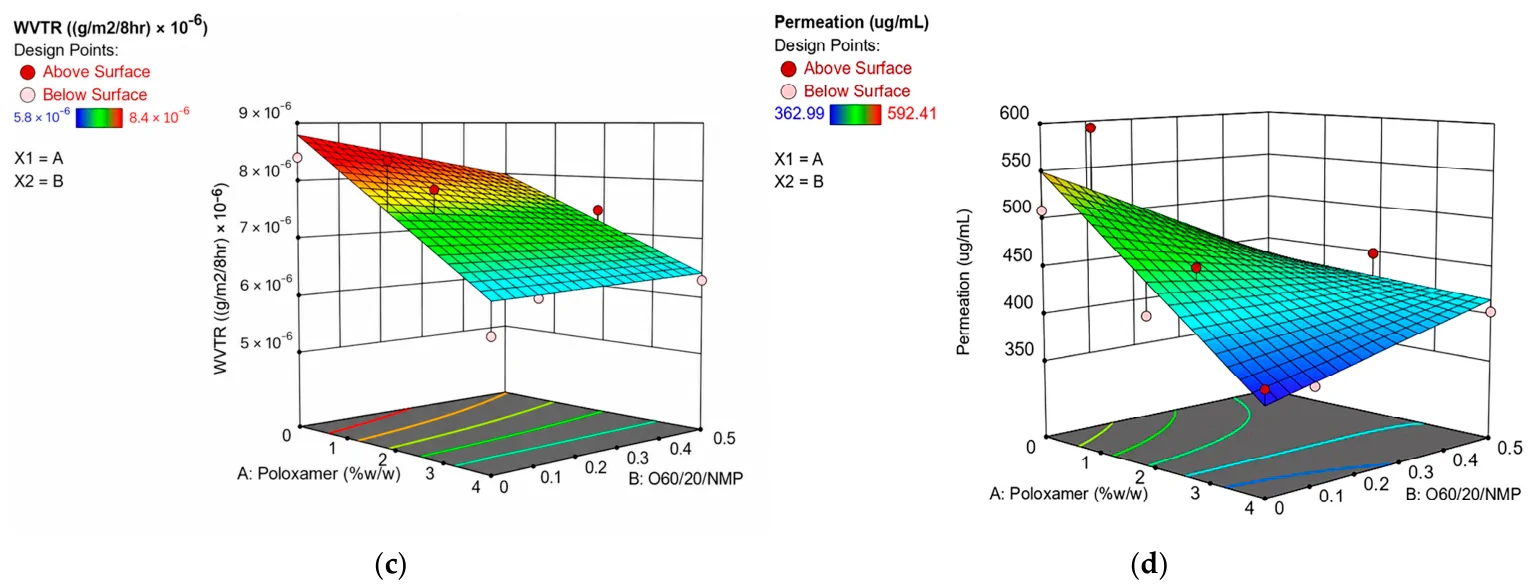
A 3D response surface plot of film performance parameters as functions of P407 and O60/20/NMP concentration (%*w*/*w*). (**a**) Drying time, (**b**) Elongation at break, (**c**) WVTR, (**d**) DHM permeation.

Positive coefficients indicate a promoting effect, while negative coefficients indicate an inhibitory effect on the respective response. Increasing P407 prolonged drying time, reduced WVTR, and decreased drug permeation, while its effect on elongation at break was not statistically significant. Increasing O60/20/NMP shortened drying time but had no significant effect on WVTR, permeation, or elongation at break. No factor significantly affected elongation at break as shown in Figure 2. Although P407 reduced WVTR, it had unfavorable effects on drying time and drug retention, the two responses most heavily weighted in the desirability optimization. Consequently, the desirability function converged on the lower boundary of the tested range (0%*w*/*w*), indicating that P407 offered no net advantage for this formulation despite its inclusion as a candidate viscosity-modifying agent. The optimized formulation was predicted to contain 0.00%*w*/*w* P407 and 0.079%*w*/*w* O60/20/NMP, as summarized in Table 3, and was selected for further characterization such as occlusive factor, moisture content, moisture uptake, swelling index, viscosity, surface morphology and drug release kinetics. The optimized formulation contained only 0.075%*w*/*w* NMP, and NMP was intended to be used as a co-solvent to enable the dissolution of the O60/20 polymer system. Applying the benchmark margin of exposure of 30 used by the U.S. Environmental Protection Agency to define a threshold of no unreasonable risk, the corresponding maximum acceptable exposure level is approximately 0.9–1.6 mg/kg-bw/day [41]. The worst case estimated exposure from the present formulation (0.0375 mg/kg-bw/day) is 24- to 43-fold below the threshold. This substantial margin indicates that NMP at 0.075%*w*/*w* in this formulation is unlikely to pose a risk of reproductive or developmental toxicity. Nevertheless, NMP is currently classified as a prohibited substance under the EU Cosmetics Regulation [42] and under the ASEAN Cosmetic Directive Annex II [43], irrespective of the low exposure margin calculated above. Notably, DMSO, which was intentionally avoided in the present formulation, is likewise listed under ASEAN Cosmetic Directive Annex II [43]. The substitution of NMP with a cosmetically compliant carrier solvent for O60/20 therefore remains a necessary step toward establishing this system’s viability for regulatory-compliant topical or cosmetic application, and the present findings should accordingly be regarded as a preliminary topical delivery platform rather than a cosmetic-ready formulation.

### 3.3. In Vitro Drug Permeation Study of the Optimized FFE

The skin permeation of DHM in FFE was evaluated and compared with DHM in FFS, oil mixture, and emulsion system and intact DHM. Strat-M^®^ membrane, a synthetic membrane developed to mimic human skin, was employed as a skin model. It is a multi-layer artificial membrane that possesses a tight top layer coated with a lipid blend resembling the lipid chemistry of the human stratum corneum (SC) and a porous lower layer resembling the epidermis and dermis layers [44].

**Figure 3:**
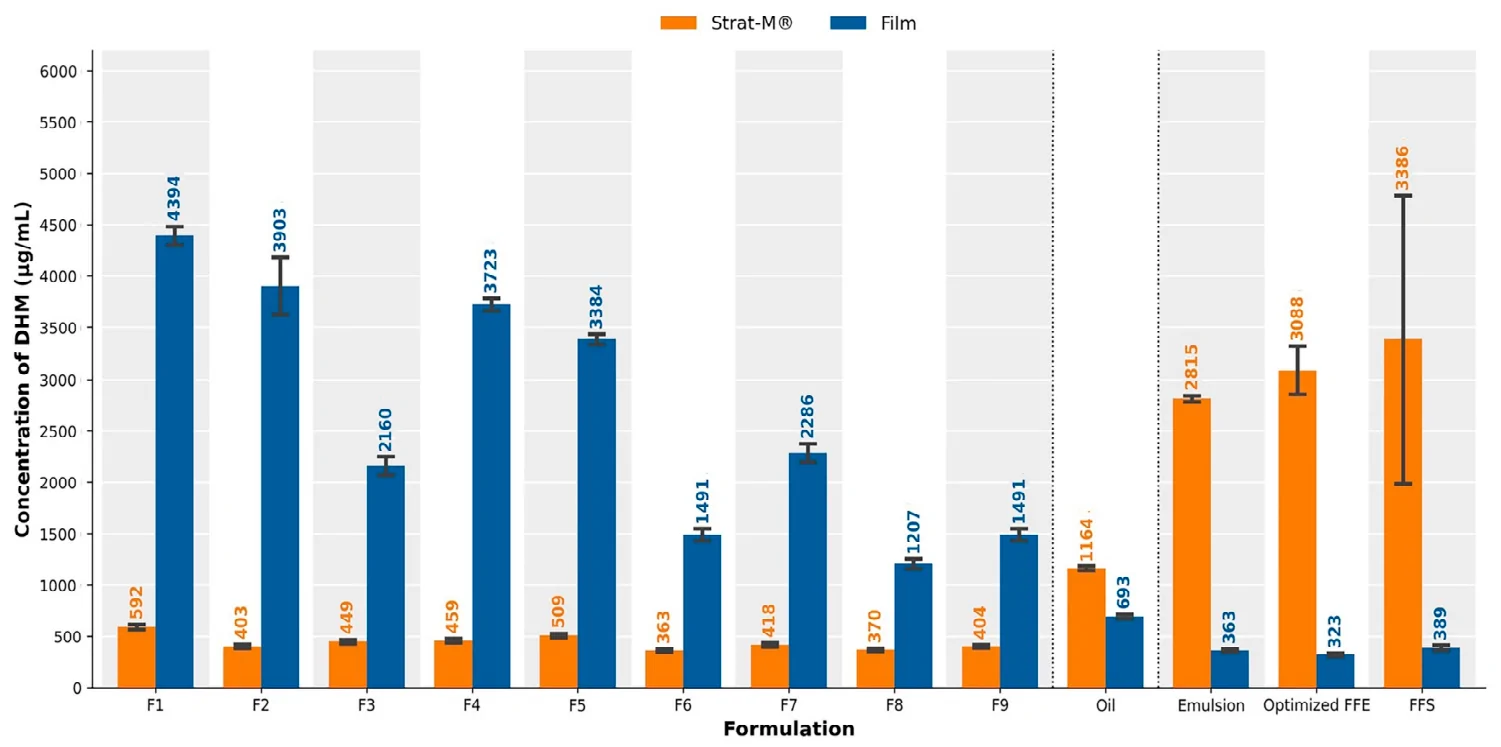
The concentration of DHM retained in the Strat-M^®^ membrane and film after 8 h of the permeation study.

Consistent with the DoE screening formulations, no DHM peak was detected in the receptor medium for the oil, emulsion, optimized FFE, and FFS systems at any time point, indicating negligible transdermal permeation across all tested systems. The DHM quantified in Figure 3 therefore represents drug retained within the dermal-equivalent Strat-M^®^ membrane and residual drug in the applied film, rather than drug that reached the systemic compartment.

The optimized FFE, FFS, and emulsion system exhibited substantially higher DHM accumulation in the membrane compared with the oil system alone. The FFS exhibited the highest membrane retention (3385.71 µg/mL), followed by the optimized FFE (3088.24 µg/mL) and the emulsion system (2814.64 µg/mL). In contrast, the oil system showed considerably lower DHM membrane retention (1164.14 µg/mL), as shown in Figure 3. When compared with the unoptimized FFE formulation (F1–F9), the concentration of DHM in these formulations was found to be only 4.84–7.90% (362.99–592.41 µg/mL) in the Strat-M^®^ membrane. These differences reflect the significance of formulation components in the optimization of the FFE, particularly the reduction of P407, a hydrophilic polymer, and the incorporation of O60/20/NMP at optimized concentrations, which were effective in enhancing drug–membrane partitioning performance.

Among the film-forming systems, the FFS exhibited the highest membrane retention. This may be attributed to the absence of the oil phase, which allows DHM to reside directly within the hydrophobic polymer matrix at a higher effective concentration gradient toward the membrane. In the Optimized FFE, while DHM retention in the membrane remained similarly high, the presence of dispersed oil droplets embedded within the polymer matrix introduced a secondary diffusion barrier, consistent with the mechanism described by Lunter and Daniels for Eudragit^®^-based film-forming emulsions, in which the oil droplets are encapsulated within a dry polymeric matrix that controls the rate of drug release to the skin [45]. The results showed that DHM membrane retention from the optimized FFE remained comparable to that of FFS, indicating that the oil phase did not negatively affect DHM delivery from the film to the skin membrane. In contrast, the oil system demonstrated the lowest DHM membrane retention, but the highest residual DHM concentration remained in the residual film. These findings could be due to the lipophilic oil phase increasing DHM solubility within the vehicle, thereby reducing its thermodynamic activity. As a result, the partitioning of the hydrophobic flavonoid out of the oil phase and into the membrane became less favorable [46].

In conclusion, the oil phase of FFE performed as a rate-controlling diffusion barrier rather than an impediment, allowing the FFE to balance sustained release with effective membrane partitioning. Overall, the optimized FFE offers a more advantageous formulation strategy than the FFS by combining comparable membrane delivery with improved drug loading and controlled-release capacity.

### 3.4. Chemical Integrity, Compatibility, and Interactions

**Figure 4:**
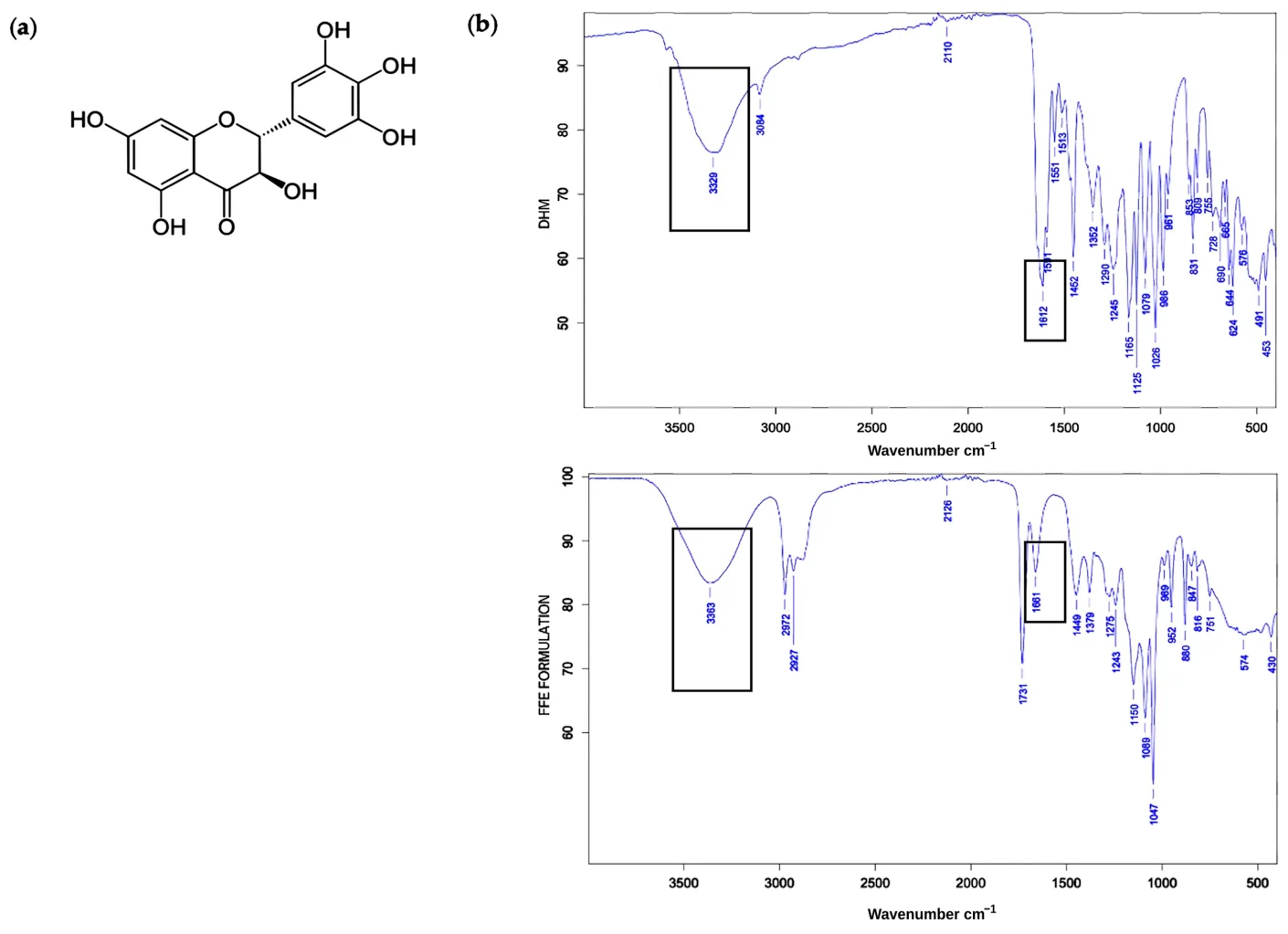
DHM chemical structure (**a**), FTIR spectra of DHM and FFE formulation (**b**).

FTIR spectroscopy was used to investigate the interactions between DHM and other components. The FTIR spectra of DHM are shown in Figure 4. DHM exhibited characteristic peaks at 3119 cm^−1^ (O-H stretching vibration), 3084 cm^−1^, and 1612–1513 cm^−1^ (aromatic ring stretching vibrations). In the FFE formulation, the characteristic peaks of DHM were retained, as shown in Figure 4. No new peaks or disappearances of characteristic peaks were detected, suggesting the absence of chemical incompatibility. These findings indicate that DHM is physically dispersed within the polymer matrix.

### 3.5. XRD Analysis

**Figure 5:**
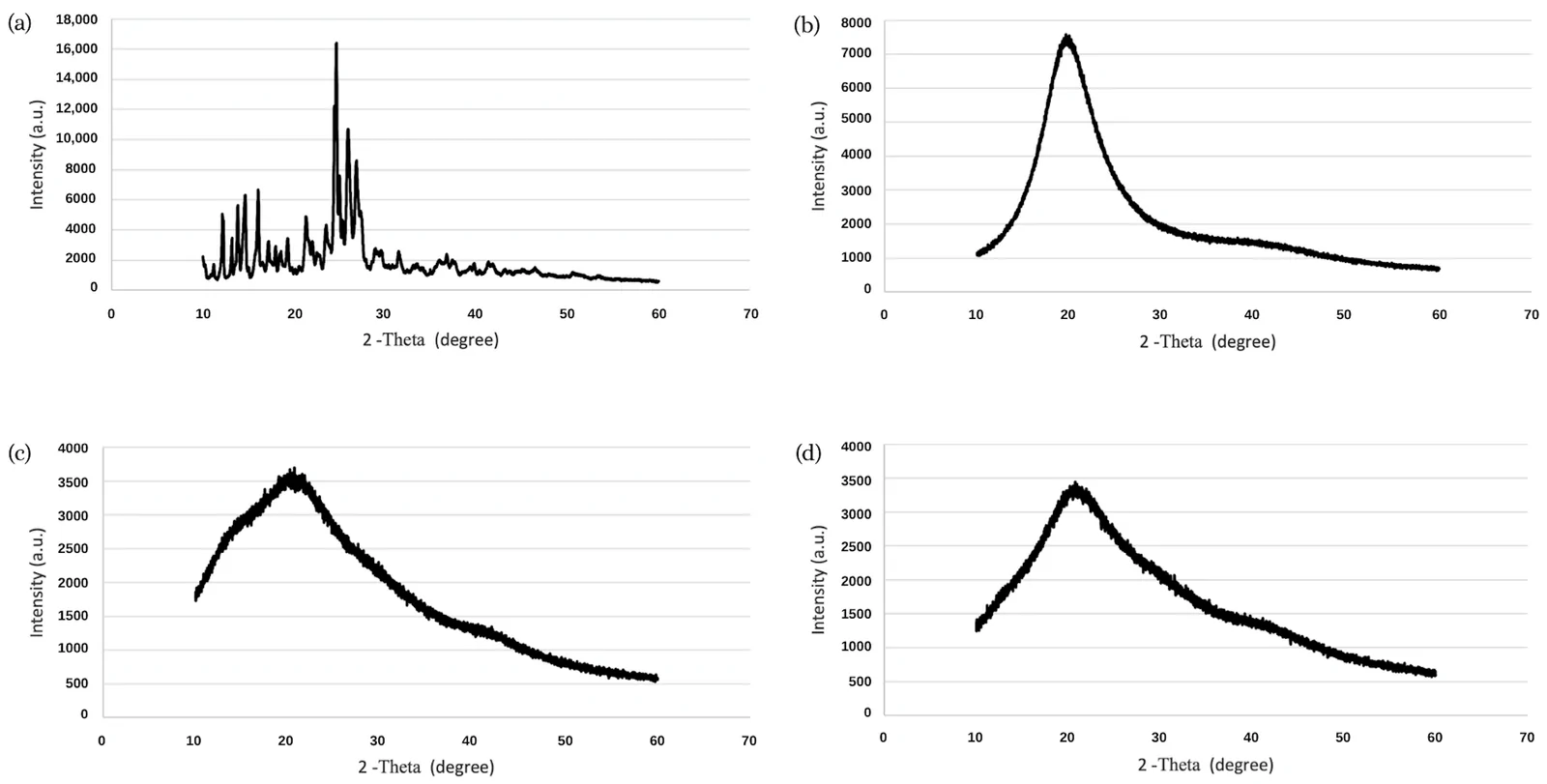
The diffractograms of (**a**) DHM, (**b**) DHM in oil phase, (**c**) FFS and (**d**) FFE formulation.

An X–ray diffraction study was performed to evaluate the crystalline structure and characteristics of pure DHM, DHM in oil phase, optimized FFE, and FFS formulation. The diffractograms are shown in Figure 5. XRD analysis demonstrated that pure DHM exhibited multiple sharp diffraction peaks, confirming its crystalline nature, as shown in Figure 5a. In contrast, the DHM-in-oil-phase system containing 0.5 g DHM in 20 g of oil (2.5%*w*/*w*) exhibited a broad halo pattern, indicating an amorphous structure, as shown in Figure 5b. After incorporation into the film-forming systems, both FFS and FFE displayed broad diffraction patterns centered around 20° with a disappearance or marked reduction of characteristic DHM peaks, as shown in Figure 5c,d. These findings suggest that DHM lost its crystalline arrangement and transitioned into an amorphous state within the polymeric matrix.

It should be noted that the FTIR and XRD data reflect the initial physicochemical state of DHM within the formulation only and do not constitute evidence of long-term chemical stability, which would require further long-term stability study.

### 3.6. Particle Size Analysis

Particle characterization was performed after diluting all formulated FFE in water to obtain 1:100 dilutions, and DHM intact in the oil phase was diluted in both water and 37%*w*/*w* ethanol to confirm the presence of nanoparticles. The particle size and size distribution of the droplets of each dilution were determined using a Zetasizer. The results, presented in Table 4, as expected, suggest that the particle size of the obtained formulations is related to the systems of the previous study [20]. The formulations F1–7, oil phase, DHM intact in oil phase, and the optimized formulation diluted in water demonstrated a small particle size of no more than 100 nm. DHM intact in oil phase and the optimized formulation showed a narrow PdI value of no more than 0.22, indicating a comparatively high uniformity of the particle size in the systems. While the particle size of DHM intact in the oil phase diluted in 37%*w*/*w* ethanol (268.33 ± 2.55) was substantially larger than that measured upon aqueous dilution (37.62 ± 0.11), this is attributed to ethanol disrupting the surfactant interfacial layer, reducing steric repulsion and promoting droplet coalescence [47]. Nevertheless, both values remained within the typical nanoemulsion droplet size range (20–300 nm) [48], suggesting that the ethanol concentration used had a slight effect on nanoemulsion droplet size.

### 3.7. Characterization and Comparison of the Optimized FFE and FFS Film Properties

#### 3.7.1. Drying Time, WVTR, Occlusive Factor, Moisture Content, Moisture Uptake and Swelling Index

The film properties of the optimized FFE and FFS were characterized and compared across multiple parameters, including drying time, WVTR, occlusive factor, moisture content, moisture uptake, and swelling index, as summarized in Table 5.

The primary film formation mechanism of *In situ* film-based Eudragit^®^ RS 100 is solvent evaporation upon application to the skin. As solvent concentration decreases, the polymer chains become entangled, leading to polymeric film formation at the target site [9]. This study found that the optimized FFE and FFS demonstrated no significant difference in the shortest drying time, falling within the desired range of ≤5 min. It was found that the 20% oil phase in FFE did not affect the drying time of the film. This might be because the oil phase in the FFE exists as nanoscale emulsified droplets (39.07 ± 0.09 nm) dispersed within the continuous phase. In film-forming emulsions, upon solvent evaporation, oil droplets become encapsulated within the dry polymer matrix while the continuous polymer–solvent phase remains the primary route through ethanol evaporation [16], and the use of volatile co-solvents such as IPA and Transcutol^®^ CG in the formulation affects the overall solvent evaporation rate, compensating for the lower concentration of ethanol used, which is advantageous as it reduces the risk of skin irritation commonly associated with high ethanol concentrations in topical formulations.

WVTR reflects the rate at which moisture passes through the formed film. A lower WVTR indicates a more occlusive film that restricts trans epidermal water loss (TEWL) [49], which is beneficial for maintaining skin hydration and promoting drug retention at the site of application. In this study, the WVTR of the optimized FFE film was higher than that of the FFS film, suggesting that the presence of the dispersed oil phase within the polymer matrix may introduce micro-channels or interfacial gaps between oil droplets and the polymer network that facilitate greater water vapor passage [50].

However, the occlusive factor of the optimized FFE film was comparable to that of the corresponding film phase, with no statistically significant difference observed (*p* = 0.68). This indicates that the barrier properties were predominantly governed by the polymer matrix, and the incorporation of the oil phase did not significantly alter the occlusive performance of the formulation.

The moisture content of the optimized FFE film was significantly higher than that of the corresponding FFS film (*p* < 0.001), while the moisture uptake of the optimized FFE film was comparable to that of the corresponding FFS film, with no statistically significant difference observed (*p* = 0.16). This difference can be attributed to oil droplets dispersed throughout the polymer network, which may create an environment that retains residual water and prevents complete solvent removal during film drying [51], resulting in a higher moisture content.

The swelling index of the optimized FFE film was significantly lower than that of the FFS film (*p* = 0.021), which may be attributed to the presence of the oil phase that reduces water penetration into the system.

#### 3.7.2. Drug Loading Capacity and Drug Content

Drug loading capacity was defined as the amount of DHM present relative to the total mass of the finished film. In this study, it also refers to DHM loading in a nanoemulsion system that disperses in the film [34]. The optimum drug loading depends on the nanoemulsion components’ ability to maximally solubilize DHM, ensuring no drug precipitation [43]. It was found that the optimized FFE provides a drug loading capacity of 0.74 ± 0.00%. This value closely matches the theoretical drug loading calculated for a dry film (0.71%), confirming that DHM was successfully incorporated into the FFE matrix with adequate solubilization within the polymer–oil system and minimal loss during the film-forming process.

Drug content expresses the efficiency with which the drug was retained relative to the amount originally added [52]. In this study, the percentage of drug content was calculated as the ratio of the measured DHM mass in the extract to the theoretical DHM mass expected in a dry film. This parameter serves as an indicator of formulation accuracy and drug recovery efficiency. The drug content was found to be 103.23 ± 0.20%, which falls within the commonly accepted range of 85–115% [53].

These findings align with the FTIR and XRD results, which showed no chemical interaction between DHM and the polymers and a crystalline to amorphous transition, respectively. This supports the efficient molecular-level dispersion of DHM, consistent with the observed loading and content values.

#### 3.7.3. Viscosity Assessment

**Figure 6:**
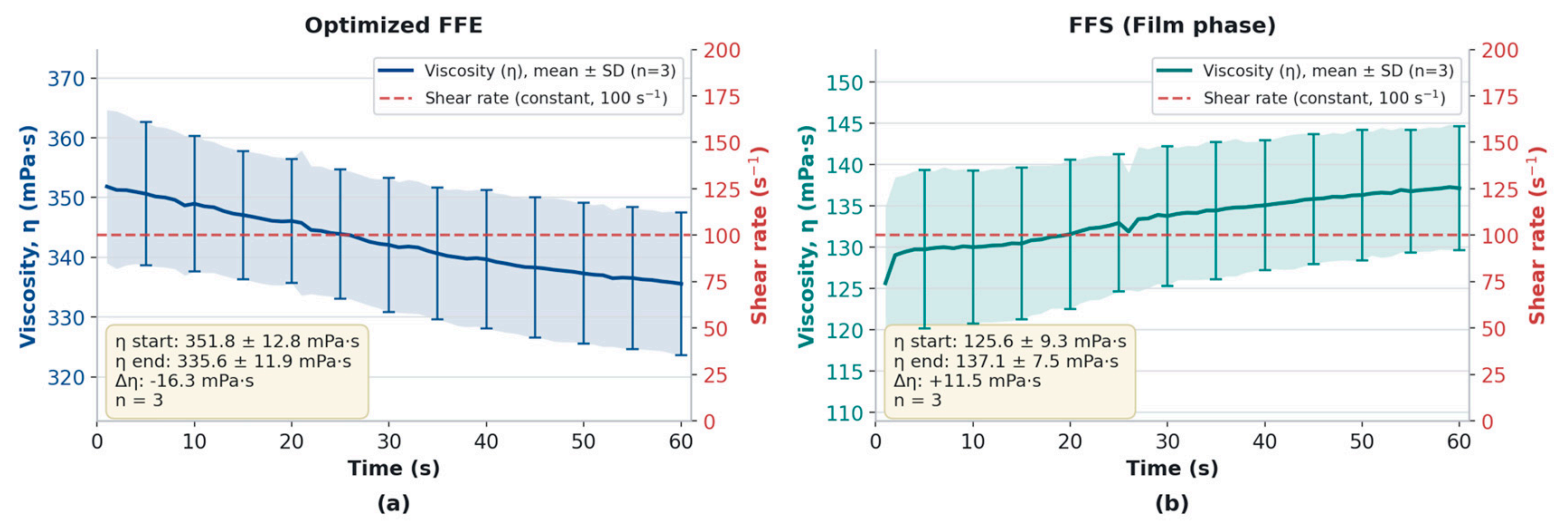
Viscosity-time profile at constant shear rate. Data presented as mean ± SD (*n* = 3). (**a**) Optimized FFE formulation, (**b**) FFS formulation.

The viscosity-time profile of the optimized FFE formulation demonstrated a gradual decrease in viscosity over time from 351.8 ± 12.8 to 335.6 ± 11.9 mPa·s (Δη = −16.3 mPa·s) under constant testing conditions at shear rate of 100 s^−1^ for 60 s, collecting 60 data points at 1 s intervals. This time-dependent viscosity decline under constant shear is consistent with thixotropic behavior, in agreement with previous report [54]: likely caused by under shear, the entangled balls change their shape and become ellipsoids. This deformation goes hand-in-hand with the increasing disentanglement of the molecules. As individual molecules have less flow resistance than entangled superstructures, the result is thixotropic flow behavior [55], The observed thixotropic softening is nonetheless a favorable characteristic for a topical vehicle, as it can facilitate spreadability during application [56]. In contrast, the FFS showed progressive viscosity increase from 125.6 ± 9.3 to 137.1 ± 7.5 mPa·s (Δη = +11.5 mPa·s) during measurement, as shown in Figure 6, indicating rheopexy or anti-thixotropic behavior. It suggests that time-dependent structural build-up is attributed to polymer chain interaction and network formation during solvent evaporation. This is generally found in the rheological testing of polymer/film-forming solution [57]. These differences indicate that the oil-containing formulation contributes to enhanced flowability and spreadability, whereas the film phase promotes structural cohesion and matrix formation.

### 3.8. Surface Morphology Evaluation of the Films

**Figure 7:**
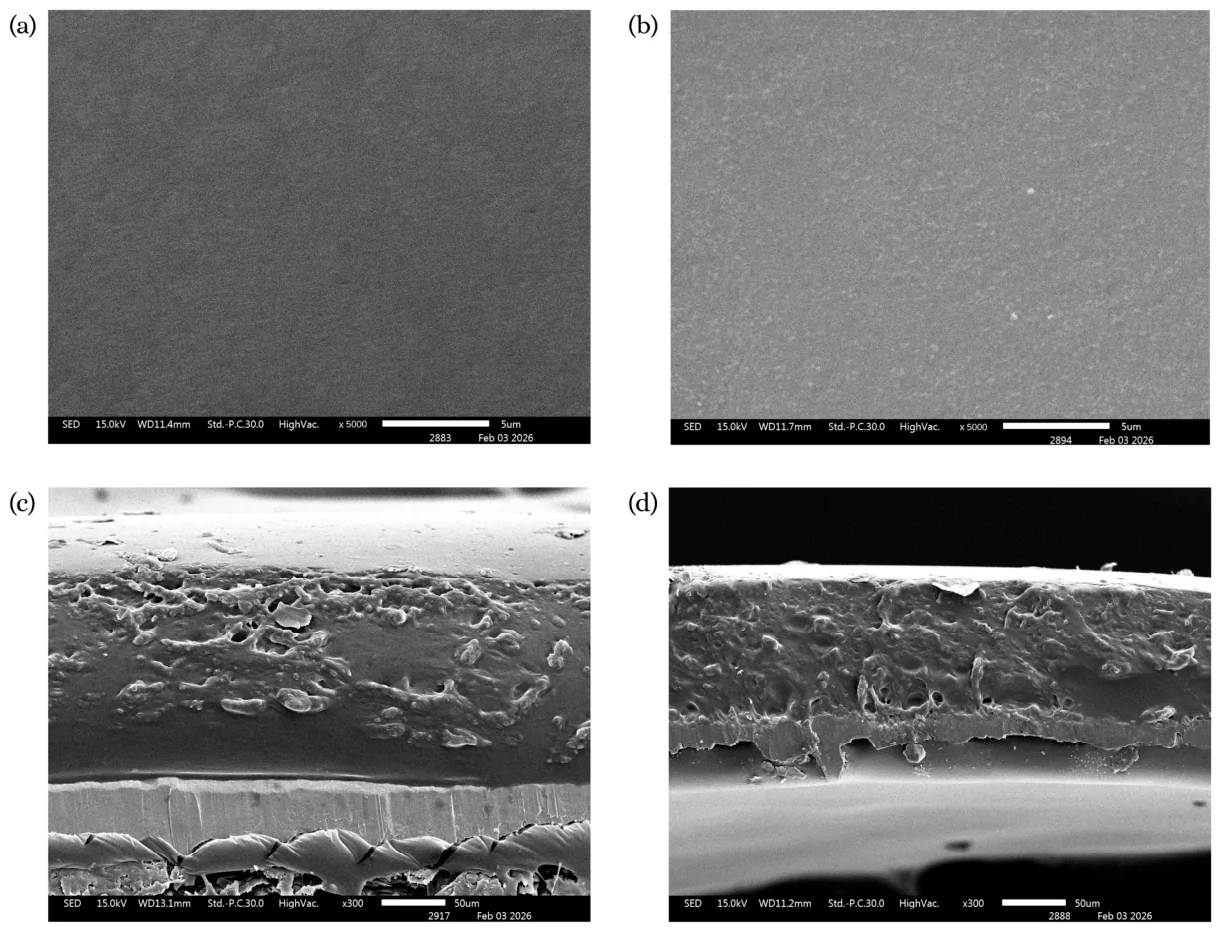
SEM micrographs of the surface of the optimized FFE film (**a**) and the film phase of the optimized FFE formulation (**b**) at 5000× magnification, and cross-sectional scanning electron micrographs of the optimized FFE film (**c**) and the film phase of the optimized FFE formulation (**d**) at 300× magnification.

Morphological investigations of the dried films obtained from optimized FFE and FFS formulations were conducted using SEM. Captured surface SEM images are depicted in Figure 7a,b for FFE and FFS, respectively, which reveals that both films exhibited a smooth texture and surface without any surface cracking or inconsistency, confirming the complete integration of the drug within the polymeric matrix [58], whereas a cross-sectional SEM image revealed that the optimized FFE exhibited a more homogeneous internal structure compared to the film phase, with porous areas observed on the external surface that resulted from solvent evaporation during film formation. This suggests solvent evaporation during film formation, as shown in Figure 7c,d, for FFE and FFS. This might be because the absence of an oil phase in FFS means that most components other than the polymer are volatile, so their rapid evaporation generates a concentration gradient within the drying film, producing a porous structure [59].

### 3.9. Drug Kinetic Release

**Figure 8:**
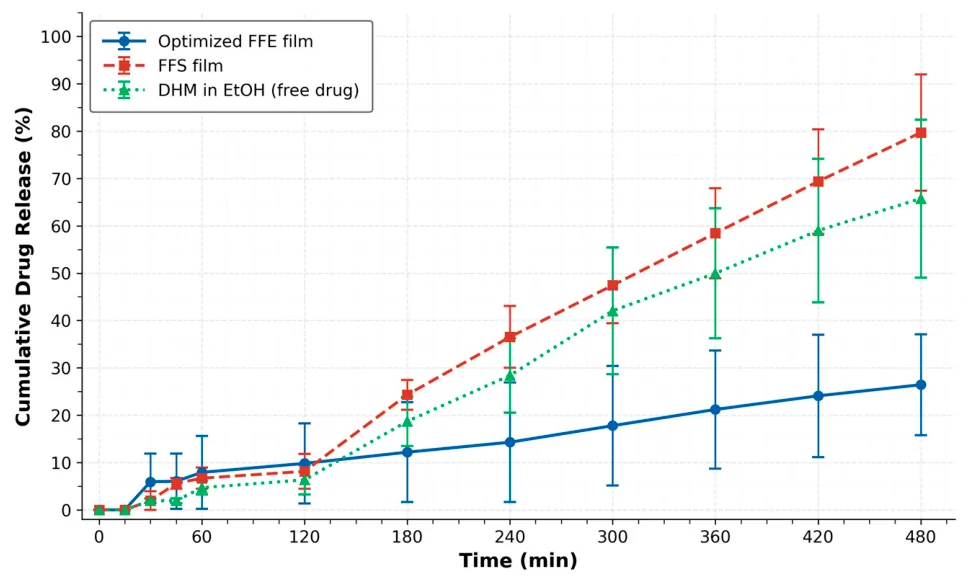
Cumulative drug release profiles of DHM from the optimized FFE film, FFS film, and free drug control (DHM in ethanol) over 480 min.

The in vitro drug release profiles of DHM from the optimized FFE film, FFS film, and the free drug control (DHM in ethanol) were evaluated over 480 min. The cumulative release profiles are shown in Figure 8, and the release mechanisms were analysed using four kinetic models following zero-order, first-order, Higuchi, and Korsmeyer–Peppas (K-P). The coefficient of determination (R^2^) for each formulation is summarized in Table 6.

As shown in Figure 8, the three formulations exhibited release behaviors over the 480-min study period. The free DHM in ethanol showed minimal initial release (<10%) within the first 120 min, followed by a sustained release at a constant rate in the absence of an initial burst effect, reaching approximately 66% cumulative release by 480 min. The FFS film exhibited an initial lag phase (0–120 min) followed by a burst release between 120 and 180 min, after which release continued at a slower, comparatively linear rate through 480 min. In contrast, the optimized FFE film demonstrated a gradual release that showed a progressive increase in cumulative drug release throughout the entire 480-min period, without an observable burst–release phase, indicating a sustained release profile. The absence of burst–release in the optimized FFE film is advantageous for the topical delivery of DHM, as it ensures a prolonged, uniform drug supply to the skin surface, supports sustained activity, and reduces the risk of local irritation associated with abrupt drug exposure [60].

Based on the highest R^2^ value, the zero-order model best described DHM release from the ethanolic solution and FFS film. Although the zero-order model provided the best overall statistical fit for FFS film (R^2^ = 0.989), this may reflect the compensating effect of the early lag and burst phases rather than genuine constant-rate release as observed from the release profile. The release mechanism of the FFS film is therefore more accurately described as biphasic, with a K-P diffusional exponent (R^2^ = 0.919). As both FFS and FFE formulations were evaluated as thin polymeric films, the K-P exponent was interpreted according to the release-exponent thresholds established for thin-film geometry, where *n* ≤ 0.5 indicates Fickian diffusion, 0.5 < *n* < 1.0 indicates anomalous (non-Fickian) transport, *n* = 1.0 indicates Case-II transport, and *n* > 1.0 indicates Super Case-II transport [61]. Thus, the FFS exponent (*n* = 1.274) corresponds to Super Case-II transport, indicating a relaxation/erosion-dominated release mechanism by the film matrix. Eudragit^®^ RS 100-based polymer matrix contributes a constant, time-independent release phase. However, the burst release observed at 120–180 min suggests that the initial rapid swelling of the polymer matrix contributes to an early disproportionate drug release before the zero-order mechanism becomes dominant [62]. It should be noted that the cellulose membrane used in this assay served as an inert support membrane rather than a skin-mimicking barrier. Its large, hydrophilic pores impose minimal resistance to drug diffusion, allowing the release rate to be governed predominantly by the physicochemical properties of the formulation rather than by the membrane itself. However, membranes have been reported to become rate-limiting when the intrinsic release rate of the drug from the donor formulation exceeds its diffusion rate across the membrane, in which case the measured profile reflects membrane permeation rather than true formulation-controlled release kinetics [63].

In the present study, the use of an inert, low-resistance cellulose membrane was intended to minimize this risk and ensure that the observed release profiles predominantly reflect formulation-controlled behaviour.

The optimized FFE film, which incorporates the oil phase within the polymer matrix, exhibited a markedly different release profile compared with both DHM in ethanol and the film phase. The optimized formulation was best described by the Higuchi model, followed by the K-P model (*n* = 0.444) with Fickian diffusion. This indicates that the matrix-like system of oil droplets immobilized by insoluble polymers is rate-controlling, as the same result was observed for the film-forming emulsion containing Eudragit^®^ RS [45,64,65]. The poor first-order fit indicates that release from the optimized formulation is not concentration-dependent, and the reduced zero-order fit compared to FFS film confirms that the oil phase alters the matrix release mechanism. This mechanistic distinction is further supported by the swelling behaviour of the two systems as shown in Table 5; the lower swelling index of the optimized FFE film restricts water ingress and polymer chain relaxation, consistent with a Higuchi model, whereas the greater swelling capacity of the FFS film facilitates matrix relaxation, consistent with its K-P exponent (*n* = 1.274) profile. The Higuchi model describes drug release proportional to the square root of time. The kinetic analysis collectively demonstrates that the structural complexity of the FFE system progressively modifies the DHM release mechanism, thereby promoting sustained release.

## 4. Conclusions

This study successfully developed and characterized dihydromyricetin, or DHM-loaded *in* situ film-forming emulsion (FFE), using Eudragit^®^ RS 100 as the film-forming polymer, avoiding the use of dimethyl sulfoxide (DMSO), which is restricted in cosmetic products. However, the optimized formulation retains N-methyl-2-pyrrolidone (NMP) (0.075%*w*/*w*) as a cosolvent, which is itself prohibited under both EU Cosmetics Regulation Annex II [42] and ASEAN Cosmetic Directive Annex II [43]. Thus, the substitution of NMP with a compliant alternative therefore remains a necessary direction for future development before this formulation can be considered fully compliant for cosmetic use. The suitable formulation was optimized through a full factorial design with poloxamer 407 (P407) and O60/20/NMP as key variables. The optimized FFE formulation demonstrated desirable physicochemical properties, including rapid film formation, adequate mechanical flexibility, and consistent water vapor transmission rate. The particle size analysis of nanoemulsions obtained from the oil phase dispersed in polymer solution confirmed that the optimized FFE produced nanoscale oil droplets with a narrow size distribution, indicating a well-dispersed and uniform emulsion system.

FTIR confirmed chemical compatibility between DHM and the excipients, while XRD demonstrated a crystalline to amorphous transition of DHM within both the FFE and film-forming solution without oil phase or *in situ* film-forming system (FFS) and FFE matrices. Relative to the FFS, oil-phase incorporation significantly increased moisture content and reduced swelling index (*p* < 0.021), with occlusive factor and moisture uptake unchanged, indicating that the oil phase modifies film physical properties without compromising barrier function. The lower swelling index reflects restricted water penetration by the hydrophobic oil droplets, favoring sustained drug retention at the application site.

In vitro drug permeation study demonstrated that both FFE and FFS achieved substantially higher DHM retention within the Strat-M^®^ membrane compared to the oil system alone, confirming the superior membrane partitioning performance of film-forming systems. The FFS exhibited the highest membrane retention (3385.71 µg/mL), followed closely by the optimized FFE (3088.24 µg/mL), suggesting that the oil phase does not negatively affect drug delivery into the membrane. Although FFS exhibited slightly higher Strat-M^®^ membrane permeation than the optimized FFE, FFE was superior to FFS based on its sustained, burst-free release profile, reduced swelling index, and its capacity to incorporate a lipophilic oil phase.

Release kinetics demonstrated that FFS followed biphasic mechanism via Super Case-II relaxation-controlled release (*n* = 1.274), governed by the Eudragit^®^ RS 100 matrix, whereas FFE followed a Higuchi/Fickian diffusion-controlled mechanism (*n* = 0.444), consistent with a heterogeneous matrix in which dispersed oil droplets act as secondary diffusion barriers.

In summary, the developed FFE addressed several key challenges reported for hydrophobic polymer-based dermal delivery systems. Unlike conventional film-forming solutions, the emulsion-based design localizes DHM within the dispersed oil phase, reducing the risk of supersaturation-induced drug crystallization upon solvent evaporation, which is known to reduce bioavailability and therapeutic efficacy, a limitation widely reported for *In situ* film-forming solutions [66]. These findings establish the optimized DHM-loaded FFE as a promising topical delivery platform, combining rapid film formation, adequate barrier properties, high drug loading efficiency, and controlled release. This innovative formulation approach supports pharmaceutical development and contributes to improved topical therapeutic options. As DHM was undetectable in the receptor phase throughout the study, the developed FFE and FFS systems demonstrated dermal-targeted retention without measurable systemic absorption, a profile consistent with the intended cosmetic (non-systemic) application of DHM as a model active in this preliminary, trend-oriented formulation study. However, several limitations should be acknowledged. The optimized formulation retains NMP translation toward cosmetic application will therefore require the substitution of NMP with a compliant co-solvent. The cellulose membrane used in the drug release kinetic study did not affect the release rate, as its large, hydrophilic pores impose minimal resistance to drug diffusion. However, this was not independently verified via a blank-membrane control; therefore, a minor contribution of the membrane to the observed release profile cannot be entirely excluded. Further in vivo permeation and long-term stability studies, together with full analytical method validation, are warranted to support clinical translation.

## Figures and Tables

**Table 1:** Independent and dependent variables with their levels and constraints for DOE.

Variables	Level		
**Independent Variables**	**[−1]**	**[0]**	**[+1]**
P407 (%*w*/*w*)	0	2	4
O60/20/NMP (%*w*/*w*)	0	0.1	0.5
**Dependent Variables**		**Constraints**	
Elongation at break (%)	Preferable (>10%) [18,19]		
Drying time (min)		Minimize	
WVTR (g/m^2^/8 h)		Minimize	
Drug permeation (µg/mL)		Maximize	

**Table 2:** Composition of *In situ* film-forming system and details of optimization by DoE.

Formulations	Independent Variables		Dependent Variables			
	P407 (%*w*/*w*)	O60/20/NMP (%*w*/*w*)	Drying Time (min)	Elongation at Break (%)	WVTR (g/m^2^/8 h) × 10^−6^	Drug Permeation (µg/mL)
1	0.00	0.10	2.97 ± 0.65 ^a,b,c^	112.88 ± 2.79 ^a^	8.40 ± 2.41 ^a^	592.41 ± 12.04 ^e^
2	4.00	0.50	3.84 ± 0.35 ^b,c,d^	117.93 ± 1.50 ^a^	7.88 ± 0.00 ^a^	402.64 ± 4.60 ^b^
3	2.00	0.50	3.30 ± 0.08 ^a,b,c^	121.22 ± 1.96 ^a^	7.35 ± 0.91 ^a^	448.65 ± 4.58 ^c^
4	2.00	0.10	3.43 ± 0.11 ^a,b,c^	117.33 ± 7.34 ^a^	7.87 ± 1.58 ^a^	459.29 ± 13.57 ^c^
5	0.00	0.00	3.71 ± 0.59 ^b,c,d^	119.87 ± 5.04 ^a^	8.40 ± 1.82 ^a^	508.58 ± 2.58 ^d^
6	4.00	0.10	4.24 ± 0.82 ^c,d^	111.73 ± 1.30 ^a^	6.30 ± 1.58 ^a^	362.99 ± 1.16 ^a^
7	2.00	0.00	4.21 ± 0.28 ^c,d^	116.68 ± 4.99 ^a^	8.40 ± 0.91 ^a^	418.17 ± 11.68 ^b^
8	4.00	0.00	5.21 ± 0.84 ^d^	111.63 ± 5.94 ^a^	5.77 ± 0.91 ^a^	370.31 ± 2.38 ^a^
9	0.00	0.50	2.84 ± 0.53 ^a,b,c^	122.02 ± 3.28 ^a^	7.87 ± 0.00 ^a^	404.66 ± 1.59 ^b^

Differences in lowercase letters indicate statistically significant differences among groups within each experiment as determined by one-way ANOVA with Tukey’s post hoc test (*p* < 0.05).

**Table 3:** The composition of the optimized FFE formulations.

	Oil Phase (20%*w*/*w*)							
	DHM	MCT		Maisine^®^ CC		Eumulgin^®^ CO		Ethanol
**Optimized FFE**	0.50	3.90		5.85		7.80		1.95
	**Film phase (80%*w*/*w*)**							
	Eudragit^®^ RS 100	Ethanol	PG	Transcutol^®^ CG	IPA	PEG 400	PVP K30	O60/20/NMP
	25.00	37.02	5.00	1.00	7.00	1.90	3.00	0.079

**Table 4:** The effects of different concentrations of excipients in formulations on particle size and size distribution (PdI).

Formulations	Particle Size (nm)	Pdl
F1	72.29 ± 0.79 ^d,e^	0.33 ± 0.01 ^c,d,e^
F2	70.35 ± 0.96 ^c,d^	0.43 ± 0.01 ^e^
F3	93.86 ± 16.25 ^f^	0.43 ± 0.10 ^e^
F4	64.45 ± 1.26 ^b,c,d^	0.35 ± 0.01 ^c,d,e^
F5	57.29 ± 0.09 ^a,b,c,d^	0.23 ± 0.00 ^b,c^
F6	47.26 ± 0.85 ^a,b^	0.37 ± 0.04 ^c,d,e^
F7	90.67 ± 1.57 ^e,f^	0.32 ± 0.03 ^c,d,e^
F8	133.80 ± 2.25 ^g^	0.22 ± 0.11 ^c,d,e^
F9	191.70 ± 17.30 ^h^	0.39 ± 0.04 ^d,e^
Optimized FFE	39.07 ± 0.09 ^a^	0.15 ± 0.01 ^a,b^
Oil phase *	51.70 ± 0.95 ^a,b,c^	0.29 ± 0.03 ^c,d^
DHM in oil phase *	37.62 ± 0.11 ^a^	0.08 ± 0.01 ^a^
DHM in oil phase in 37% ethanol	268.33 ± 2.55 ^i^	0.09 ± 0.02 ^a^

* The composition of excipients in the oil phase was the same as the optimized FFE formulation. Differences in lowercase letters indicate statistically significant differences among groups within each experiment as determined by one-way ANOVA with Tukey’s post hoc test (*p* < 0.05).

**Table 5:** The film properties of the optimized FFE and FFS.

Formulations	Film Properties					
	Drying Time (min)	WVTR (g/m^2^/8 h) × 10^−6^	Occlusive Factor	Moisture Content (%)	Moisture Uptake (%)	Swelling Index
Optimized FFE	2.68 ± 0.30	7.87± 2.73	41.07 ± 6.41	26.63 ± 2.68	15.93 ± 2.62	48.53 ± 2.08
FFS	2.19 ± 0.11	2.10 ± 0.91	42.86 ± 3.09	12.81 ± 0.75	18.74 ± 1.05	155.37 ± 22.22

**Table 6:** Kinetics of DHM release models from optimized FFE, FFS, and intact DHM in ethanol.

Formulation	Zero Order		
	k		R^2^
Optimized FFE	0.0733		0.900
FFS	0.171		0.989
Intact DHM/Ethanol	0.146		0.985
Formulation	**First order**		
	**k**		**R^2^**
Optimized FFE	2.4 × 10^−3^		0.538
FFS	3.9× 10^−3^		0.861
Intact DHM/Ethanol	4 × 10^−3^		0.902
Formulation	**Higuchi**		
	**k**		**R^2^**
Optimized FFE	1.81		0.970
FFS	3.86		0.894
Intact DHM/Ethanol	3.28		0.884
Formulation	**Korsmeyer–Peppas**		
	**n**	**k**	**R^2^**
Optimized FFE	0.444	0.558	0.941
FFS	1.274	0.809	0.919
Intact DHM/Ethanol	1.403	0.805	0.859

## Data Availability

The data underlying this study are available in the published article.

## Abbreviations

The following abbreviations are used in this manuscript:

ATR	Attenuated total reflectance
DMSO	Dimethyl sulfoxide
DNA	Deoxyribonucleic acid
DNMT1	DNA methyltransferase 1
DoE	Design of experiment
FFE	*In situ* film-forming emulsions
FFS	*In situ* film-forming system
FTIR	Fourier transform infrared spectrometer
HPLC	High-performance liquid chromatography
HPMC	Hydroxypropyl methylcellulose
IPA	Isopropyl alcohol
K-P	Korsmeyer–Peppas
NMP	N-methyl-2-pyrrolidone
O60/20	Octyl cyanoacrylate
P407	Poloxamer 407
PBS	Phosphate-buffered saline
PEG	Polyethylene glycol
PEG400	Polyethylene glycol 400
PVP	Polyvinyl pyrrolidone
RH	Relative humidity
SC	Stratum corneum
WVTR	Water vapor transmission rate
XRD	X–ray diffraction
